# Bikinin-like inhibitors targeting GSK3/Shaggy-like kinases: characterisation of novel compounds and elucidation of their catabolism *in planta*

**DOI:** 10.1186/1471-2229-14-172

**Published:** 2014-06-19

**Authors:** Wilfried Rozhon, Wuyan Wang, Franz Berthiller, Juliane Mayerhofer, Tingting Chen, Elena Petutschnig, Tobias Sieberer, Brigitte Poppenberger, Claudia Jonak

**Affiliations:** 1 GMI-Gregor Mendel Institute of Molecular Plant Biology, Austrian Academy of Sciences, Vienna Biocenter, Dr. Bohr-Gasse 3, Vienna 1030, Austria; 2 Biotechnology of Horticultural Crops, Technische Universität München, Liesel-Beckmann-Straße 1, Freising 85354, Germany; 3 Max F. Perutz Laboratories, Department of Microbiology, Immunobiology and Genetics, University of Vienna, Vienna 1030, Austria; 4 Present address: Plant Biochemistry, ETH Zürich, Universitätsstr. 2, Zürich 8092, Switzerland; 5 Center for Analytical Chemistry, Department of Agrobiotechnology, University of Natural Resources and Life Sciences, Konrad Lorenz Straße 20, Tulln 3430, Austria; 6 Present address: Albrecht-von-Haller-Institute of Plant Sciences, Department of Plant Cell Biology, Georg-August-University Göttingen, Julia-Lermontowa-Weg 3, Göttingen 37077, Germany; 7 Department of Plant Sciences, Research Unit Plant Growth Regulation, Technische Universität München, Liesel-Beckmann-Straße 1, Freising-Weihenstephan 85354, Germany

**Keywords:** Brassinosteroid, GSK-3/shaggy-like kinase, Inhibitor, Protein phosphorylation, Signal transduction

## Abstract

**Background:**

Plant GSK-3/Shaggy-like kinases are key players in brassinosteroid (BR) signalling which impact on plant development and participate in response to wounding, pathogens and salt stress. Bikinin was previously identified in a chemical genetics screen as an inhibitor targeting these kinases. To dissect the structural elements crucial for inhibition of GSK-3/Shaggy-like kinases by bikinin and to isolate more potent compounds we synthesised a number of related substances and tested their inhibitory activity *in vitro* and *in vivo* using *Arabidopsis thaliana*.

**Results:**

A pyridine ring with an amido succinic acid residue in position 2 and a halogen in position 5 were crucial for inhibitory activity. The compound with an iodine substituent in position 5, denoted iodobikinin, was most active in inhibiting BIN2 activity *in vitro* and efficiently induced brassinosteroid-like responses *in vivo*. Its methyl ester, methyliodobikinin, showed improved cell permeability, making it highly potent *in vivo* although it had lower activity *in vitro*. HPLC analysis revealed that the methyl residue was rapidly cleaved off *in planta* liberating active iodobikinin. In addition, we provide evidence that iodobikinin and bikinin are inactivated *in planta* by conjugation with glutamic acid or malic acid and that the latter process is catalysed by the malate transferase SNG1.

**Conclusion:**

Brassinosteroids participate in regulation of many aspects of plant development and in responses to environmental cues. Thus compounds modulating their action are valuable tools to study such processes and may be an interesting opportunity to modify plant growth and performance in horticulture and agronomy. Here we report the development of bikinin derivatives with increased potency that can activate BR signalling and mimic BR action. Methyliodobikinin was 3.4 times more active *in vivo* than bikinin. The main reason for the superior activity of methyliodobikinin, the most potent compound, is its enhanced plant tissue permeability. Inactivation of bikinin and its derivatives *in planta* involves SNG1, which constitutes a novel pathway for modification of xenobiotic compounds.

## Background

**Figure 1:**
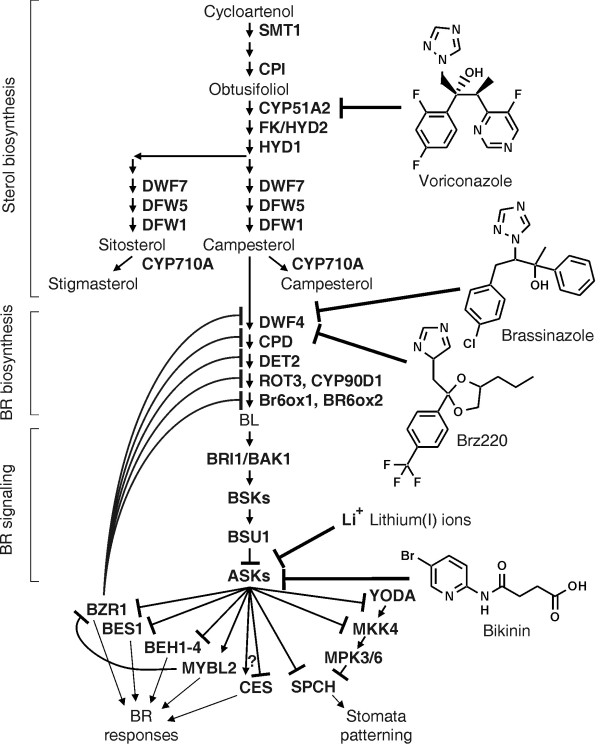
Targets of inhibitors interfering with sterol biosynthesis, BR biosynthesis and BR signal transduction.

Brassinosteroids (BRs) are plant steroid hormones involved in many processes including cell expansion and division, pollen tube growth, vascular tissue development, senescence and modulation of stress responses [1]. Plants deficient in brassinosteroids display characteristic phenotypes including severe dwarfism, shortened hypocotyls and round shaped dark green leaves. An analysis of *Arabidopsis thaliana* mutants showing such phenotypes revealed a number of enzymes crucial for production of BRs. Depending on the affected pathway these enzymes can be divided into two groups: the first is involved in general sterol biosynthesis (Figure 1) and includes DWF5 [2], STE1/DWF7 [3,4] and DWF1/CBB1 [5,6]. The second group includes DWF4 [7], CPD [8], DET2 [9], ROT3, CYP90D1 [10], BR6ox1 and BR6ox2 [11]. These enzymes are involved in the BR biosynthesis pathway that starts from the bulk sterol campesterol as a precursor and ultimately yields brassinolide (BL), the most active BR (Figure 1). The expression of most enzymes of the BR synthesis pathway is negatively regulated by BR signalling while transcript levels of enzymes involved in general sterol biosynthesis are not BR responsive.

BL is perceived by the receptor kinase BRI1 [12] and its co-receptor BAK1 [13,14], which, unlike animal steroid receptors, localise to the cell membrane. The signal is transduced by the BSK group of receptor-like cytoplasmic kinases [15] and the phosphatase BSU1 [16] to ASKs, *A. thaliana* GSK-3/Shaggy-like kinases, which are inactivated in response to BL. ASKs are a family of serine/threonine protein kinases that can be grouped into four classes [17]. Several ASKs are involved BR signalling [16,18-21], some ASKs have been shown to play a role in stress responses [22,23]. In the absence of BL the ASKs are active and can phosphorylate a number of transcription factors including BES1 [24], BZR1 [25] and their homologues BEH1 to BEH4 [26], MYBL2 [27], SPCH [28] and presumably also CES [29]. Interestingly, BIN2 (ASKη) and some other ASKs can also phosphorylate and thereby inactivate YODA [30] and MKK4 [31], two protein kinases acting in the MAP-kinase cascade that regulates SPCH activity. Similar to YODA and MKK4 most transcription factors are inhibited by BIN2-mediated phosphorylation. For instance, BES1 and BZR1 can only bind DNA in their unphosphorylated form to regulate gene expression [21].

Although a number of enzymes involved in sterol and BR synthesis and BR signalling are known, specific inhibitors are available only for a few of them (Figure 1). Recently, the triazole derivative voriconazole was shown to be a potent and specific inhibitor of plant CYP51s. Plants treated with this compound showed significantly reduced sterol and brassinosteroid levels and exhibited the typical signs of BR deficiency [32].

The observation that the gibberellic acid biosynthesis inhibitor uniconazole has a slight inhibitory effect on brassinosteroid biosynthesis led to the development of brassinazole [33] and Brz220 [34], two triazole derivatives (Figure 1) that target the heme iron of cytochrome P450 monooxygenase DWF4 [35,36]. Brassinazole has widely been used to study the synthesis and effects of brassinosteroids [37-41]. Furthermore, brassinazole was employed in genetic screens to isolate mutants that do not respond to this compound, which enabled the identification of the transcription factor BZR1 [25]. Several other inhibitors of sterol/BR biosynthesis are also known including Brz2001 [42], propiconazole [43], ketoconazole [44] and itraconazole [32]. However their molecular targets have remained elusive.

High concentrations of lithium ions (~10 mM) are used to inhibit the mammalian kinase GSK3β [45,46] and plant ASKs [47-49]. However, lithium(I) lacks specificity [50] and induces severe ion toxicity in plants [51-53], thus limiting its value for *in vivo* studies. Bikinin was identified by a chemical genetics approach as a compound that mimics BL treatment [54]. Bikinin is a non-steroidal compound that acts as an ATP-competitive inhibitor for plant GSK-3/Shaggy-like kinases and thereby induces constitutive brassinosteroid responses. Bikinin is the monoamide of succinic acid with 2-amino-5-bromopyridine. The bromine in position 5 of the pyridine ring and the carboxylic acid group were recognised as being important for its activity [54]. However, a more detailed structure-activity relationship study to reveal the contribution of specific structural elements, for instance length of the aliphatic side chain and position of the heterocyclic nitrogen, is lacking and preliminary data indicated that substitution of bromine by other halogens might lead to more active compounds [54].

Here we studied the structural elements necessary for bikinin activity in more detail, which allowed us to isolate derivatives with enhanced activity and, importantly, increased cell-permeability. Moreover, we present evidence for the catabolic fate of bikinin and its derivatives after cellular uptake and identify a novel pathway for modification of xenobiotic compounds.

## Results

### Synthesis

To gain more insight into the structural elements important for bikinin activity and to further improve bikinin inhibitor potency, we synthesised a set of bikinin derivates. Bikinin and other derivatives were prepared by formation of amides from substituted aminopyridines and cyclic carboxylic acid anhydrides or chlorides of dicarboxylic acid monomethyl esters (Additional file 1). In the last case the methyl group was subsequently removed by alkaline hydrolysis, if required. Determination of the pK_a_ of selected compounds revealed that they are weak acids (Additional file 1).

### *In vitro* inhibitor potency

**Figure 2:**
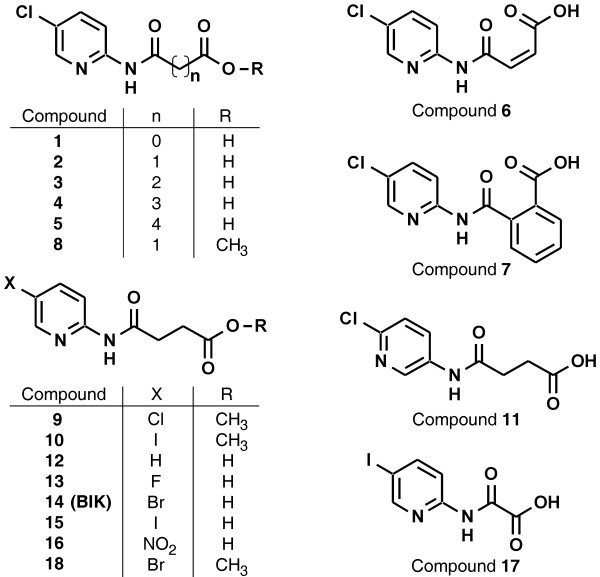
**Structures of the synthesised compounds.** The compounds 14 and 15 are also called bikinin (BIK) and iodobikinin, respectively. The compounds 18 and 10, also called methylbikinin and methyliodobikinin, respectively, represent their methylesters.

**Figure 3:**
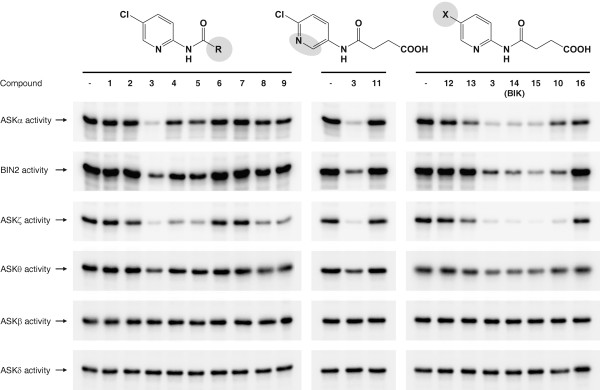
***In vitro *inhibitor activity.** For ASK *in vitro* kinase assays, purified GST-ASK fusion proteins were incubated with MBP as a substrate and [γ-^32^P]-ATP as a co-substrate in absence (−) or presence of 10 μM of the different compounds (the numbers correspond to Figure 2). Compounds 1 to 9 (left panel) differ in the aliphatic side chain. The influence of the position of the heterocyclic nitrogen was tested with compounds 3 and 11 (middle panel; the molecular structure shown represents compound 11). The right panel shows the effect of the halogen substituent of the pyridine ring. BIK, bikinin.

Bikinin is a potent inhibitor of group I and group II ASKs. ASKθ, a group III ASK is moderately inhibited. The second kinase of this class, ASKβ, and the group IV kinase ASKδ are not inhibited [54]. To investigate the inhibitor activity of the set of bikinin derivates on ASK activity, representatives of all four ASK groups were expressed as recombinant GST fusion proteins in *E. coli* and the potency of the synthesised compounds (Figure 2) on the selected ASKs was assayed by *in vitro* kinase assays using the commonly used artificial substrate myeline basic protein (MBP) (Figure 3).Compounds 1 to 5 were assayed to investigate the effect of length variation of the aliphatic side chain. The most active compound, no. 3, had a chain consisting of 4 carbons (Figure 2). The glutaryl (no. 4; five carbons) and the adipoyl (no. 5; six carbons) derivatives clearly had a lower potency while the shorter derivatives (no. 1 and 2; two and three carbons, respectively) had almost no effect. Introduction of a double bond into a side chain of optimal length abolished potency completely (Figure 3, compound 6) indicating that the steric conformation is highly important. To test whether the carboxy group of the aliphatic chain is essential for activity or if an oxo group is sufficient, we included compounds 9 and 10, which are the methyl esters of compounds 3 and 15, respectively. Compound 8, a structural isomer of compound 3, was also tested (Figure 2). As shown in Figure 3, the methylated variants showed dramatically reduced inhibitory effects confirming that a terminal carboxy group is essential.

Having identified the optimal side chain, we next investigated the influence of the heterocyclic ring in more detail. Compounds 3 and 11 both have an amido succinyl side chain but differ in the position of the heterocyclic nitrogen. *In vitro* kinase assays revealed that compound 3 is more potent (Figure 3), demonstrating that the heterocyclic nitrogen must be adjacent to the position carrying the amido succinic acid substituent.

Previous data indicated that a bromine substituent on position 5 of the pyridine ring is critical for biological activity of bikinin [54]. Compounds 12 to 16 were synthesised to test the effect of other substituents. As indicated in Figure 3 the chloro, bromo and especially the iodo derivative were highly active while the fluoro compound exhibited a very low potency. The unsubstituted and the nitro derivatives were inactive. Quantification of residual kinase activities showed that the inhibitory effect on BIN2 increased with the atomic number of the halogen substituent while an opposite but less pronounced effect could be observed for ASKθ (Additional file 2).

In summary, active derivatives inhibited ASKα, BIN2 and ASKζ strongly while ASKθ was moderately inhibited and their effects on ASKβ and ASKδ were negligible. Active compounds were characterised by an aliphatic C_4_ side chain with a terminal carboxy group and a heterocyclic nitrogen next to the amido group. In addition, a halogen substituent of the aromatic system was crucial. A fluoro residue had only a slightly promotive effect while the chloro, bromo and iodo derivatives were highly active.

### *In vivo* effects of the inhibitors

**Figure 4:**
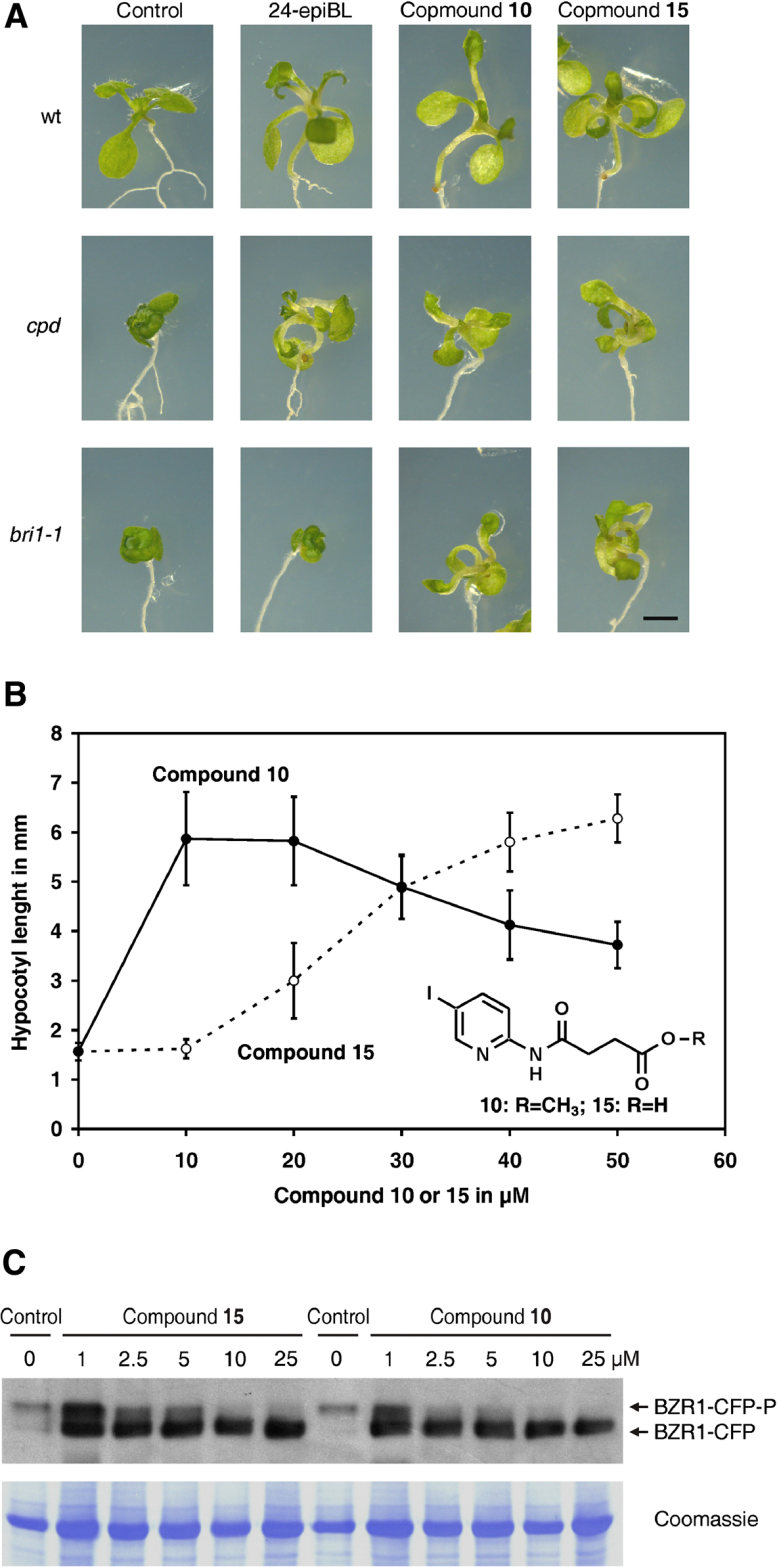
***In vivo *effects of the inhibitors. (A)** 7-day-old wild type *A. thaliana* seedlings, the brassinosteroid synthesis mutant *cpd* or the signalling mutant *bri1-1* were transferred to ½ MS medium containing 1 μM 24-epi-brassinolide (24-epi-BL), 30 μM compound 10 or 30 μM compound 15 and incubated for 7 days under long day conditions. All pictures were taken with the same magnification. The size bar represents 1 mm. **(B)** The hypocotyl length of 7-day-old *A. thaliana* Col-0 seedlings grown on ½ MS containing 1% sucrose and supplemented with compound 10 or 15 at the indicated concentrations were measured. The means and standard deviations were calculated from at least 25 seedlings. **(C)** Plants expressing BZR1-CFP were treated with the indicated compound for 1 h. Subsequently, the phosphorylation status of BZR1 was detected by western blot analysis using a GFP-antibody. A coomassie R250 stain is shown as loading control.

Brassinosteroid deficient plants are severely dwarfed and display dark green, epinastic leaves and shortened hypocotyls. Application of 24-epi-brassinolide, a synthetic brassinosteroid, can partially rescue BR biosynthesis mutants like *cpd*[8] but does not change the phenotype of BR signalling mutants like *bri1-1*[55]. In contrast, bikinin can rescue both mutants partially [54]. To investigate the *in vivo* potency of the synthesised derivatives *cpd* and *bri1-1* mutants were transferred to media supplemented with these compounds. Seedlings treated with active compounds showed expanded leaves, increased hypocotyl lengths and were light green. The potency to rescue the phenotype correlated with the results of the *in vitro* assay except for compounds 10 (Figure 4A) and 9 (data not shown), which showed little potency *in vitro* but were highly active *in vivo*. Because of this unexpected result we compared the bioactivities of compound 15, which has a free carboxy group terminal at its aliphatic side chain, and of compound 10, which is the methylated counterpart. In addition, we also synthesised compound 18, which is the methylated counterpart of bikinin (compound 14). To obtain quantitative data we measured the impact of these compounds on hypocotyl elongation of *A. thaliana*, an assay frequently used to investigate BR action [6,9,56]. The methylated compound 10 already showed a clear effect on hypocotyl length at 10 μM, while approximately 40 μM of compound 15 were necessary to obtain comparable effects (Figure 4B). At such high concentrations compound 10 already showed toxic effects that impeded full hypocotyl elongation. Similarly, methylated compounds 9 and 18 showed a much higher biological activity than their unmethylated counterparts 3 and 14 (bikinin), respectively (Additional file 3). We also compared compound 10, the most active inhibior, and bikinin (compound 14). Compound 10 and bikinin had IC_50_ values (half maximal inhibitory concentrations) of 6.9 and 23.3 μM, respectively (Additional file 3C), showing that compound 10 is 3.4 times more active in the hypocotyl eleongation assay than the original bikinin.

Next we wanted to investigate the potency of the methylated compounds at the molecular level. ASKs involved in BR signalling downregulate the activity of BES1/BZR1-family transcription factors by phosphorylation. This modification causes an electrophoretic mobility shift allowing convenient detection of *in vivo* ASK activity by western blot analysis [20,24-26,54]. *A. thaliana* seedlings expressing BZR1-CFP were treated with different concentrations of compound 10 and 15 and the BZR1 phosphorylation pattern was compared by western blot analysis with that of mock treated plants (Figure 4C). Both compounds were capable to inducing a shift of BZR1-CFP to its de-phosphorylated form. A comparable result was also obtained for plants expressing BES1-CFP (Data not shown). A similar shift was also seen in *A. thaliana* protoplasts co-transformed with constructs of CFP-tagged BZR1 and Myc-tagged ASKζ after treatment with different concentrations of compounds 10 and 15 (Additional file 4).

Thus, surprisingly, the methylated derivatives 9, 18 and 10 showed similar or even higher activities *in vivo* than their free acid counterparts 3, 14 and 15, respectively, although in *in vitro* kinase assays the methyl esters had a weaker effect on ASK activity.

### Methylated bikinin derivatives are rapidly hydrolysed *in planta*

**Figure 5:**
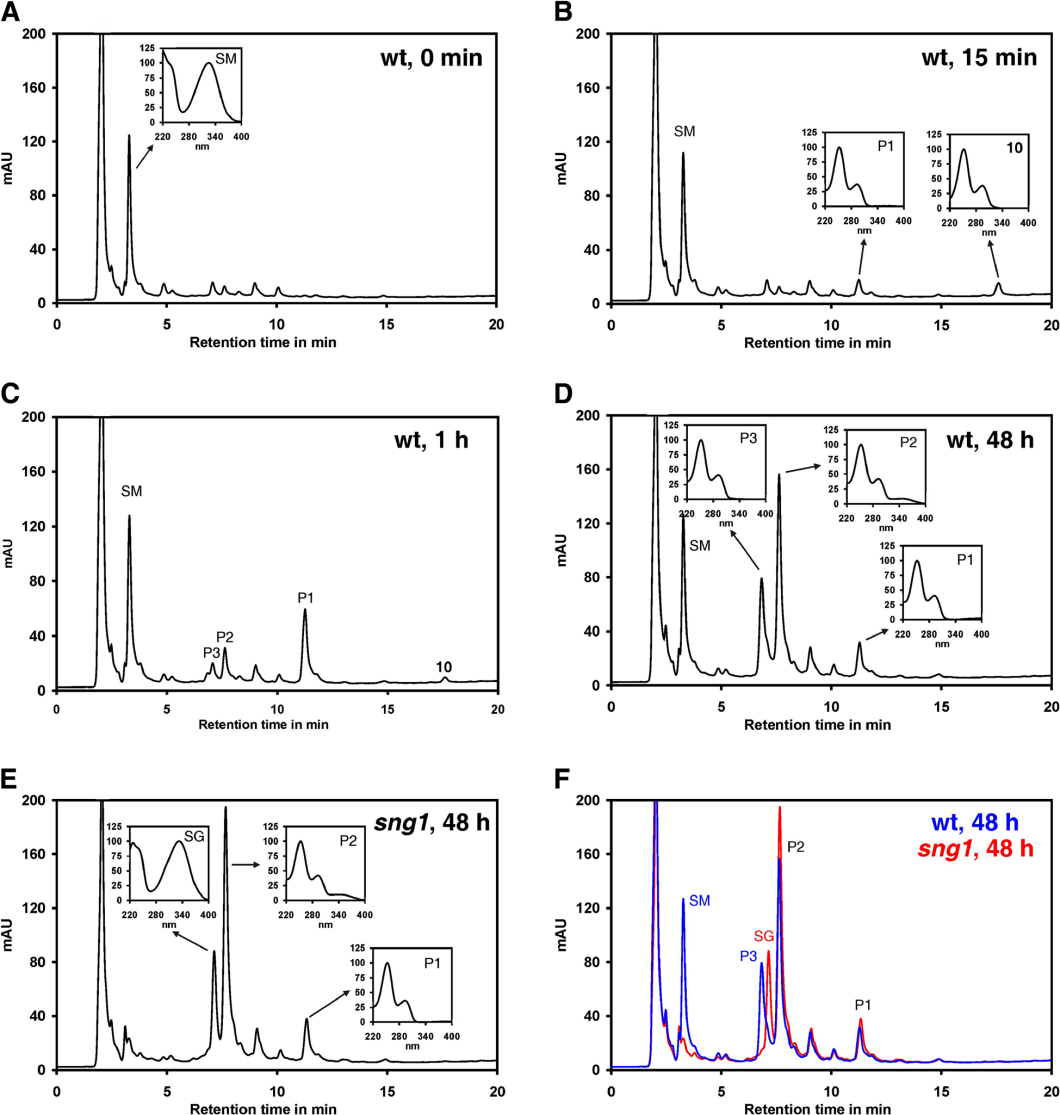
**Methylated compounds are rapidly hydrolysed *in planta*. *A. thaliana *Col-0 seedlings were infiltrated with a solution containing 50 μM compound 10. (A)** Control samples were taken before infiltration and analysed by HPLC. **(B)** Already after 15 min a second peak designated P1 appeared. **(C)** After 1 h two additional peaks, P2 and P3 were visible, **(D)** which became even more pronounced after 48 hours. **(E)** The same experiment was performed with *sng1* mutants. The chromatogram obtained with the sample taken after 48 h is shown. **(F)** Overlay of the 48 h chromatograms of the Col-0 sample and the *sng1* sample. The small boxes inserted into the chromatograms show the UV spectra of the peaks in the range of 220 to 400 nm. SM, sinapoylmalate; SG, sinapoylglucose; mAU, milli absorption units recorded at 250 nm.

To investigate these conflicting results in more detail, we analysed the fate of compound 10 *in vivo*. Seedlings were infiltrated with compound 10 and plant extracts subsequently analysed by HPLC. Only trace amounts of 10 could be observed but a novel peak, designated P1, appeared (Figures 5A and B). This peak could be identified by its retention time and its UV spectrum as compound 15 (Figure 5 and Additional file 5) indicating that compound 10 is rapidly converted to the highly active compound 15 *in planta* and explaining the different potency of compound 10 *in vitro* and *in vivo*. Prolonged incubation led to formation of two additional peaks, designated P2 and P3 (Figure 5C and B). The spectra of these two compounds indicate that they are derived from 15, most likely by modification of the aliphatic side chain. Similar results were obtained for the pairs 3/9 and 14/18 (data not shown).

### Tissue permeability of bikinin derivatives

The previous result that the methylated compounds are rapidly hydrolysed to their corresponding active free acid forms provided an explanation for the observation that the methylated compounds showed little or no activity *in vitro* while they were highly active *in vivo*. However, this result could not explain why the methylated derivatives 10, 9 and 18 showed significantly higher *in planta* activities than their free acid counterparts 15, 3 and 14, respectively (Figure 4B and Additional file 3).

**Figure 6:**
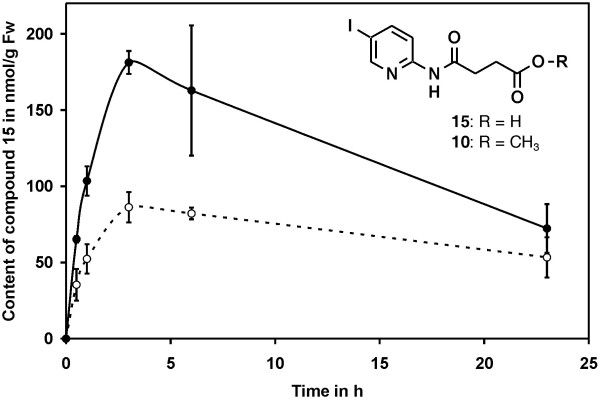
**Methylation increases tissue-permeability.***A. thaliana* seedlings were incubated in 50 μM solutions of compounds 10 and 15 in ½ MS medium. Samples were taken after the indicated time and the *in situ* levels of compound 15 were analysed by HPLC. The solid line represents the results for plants incubated with compound 10 and the dashed line shows the results for compound 15. The averages and standard deviations were calculated from 3 independent assays.

The cell-permeability of a substance is an important characteristic for its *in vivo* potency. Thus, we determined the uptake of compounds 10 and 15 by treatment of *A. thaliana* seedlings with the compounds and subsequent quantification of their internalised concentrations (Figure 6). Since compound 10 is rapidly converted to 15, only the *in situ* concentration of the cleavage product, compound 15, was measured. Uptake of both compounds was rapid and the highest *in situ* concentrations were observed after 3 hours of treatment. It is important to note that the plant internal concentrations exceeded those of the medium. While 50 μM were present in the medium, *in situ* concentrations of about 90 μM were measured after treatment with compound 15 and more than 180 μM after treatment with compound 10. These data show that the methylated inhibitor has an increased cell-permeability as compared to its unmethylated analogue, thus providing an explanation for the increased biological activity of the methylated inhibitors.

### Catabolic fate of bikinin derivatives

After prolonged inhibitor treatment the *in situ* concentration of compound 15 declined (Figure 6), concomitant with the appearance of additional peaks that had already been observed in the previous experiment (Figure 5D and data not shown). According to the retention times and UV spectra, the same end products, P2 and P3, were obtained for compound 10 and 15 (data not shown).

**Figure 7:**
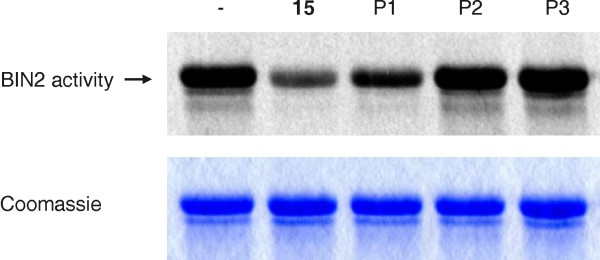
**Activity of the modified compounds.** The *in planta* modification products of compound 10 were isolated and assayed for their potency to inhibit BIN2 activity *in vitro*. Compound 15 was used as a control. Each substance was used at a concentration of 10 μM. The same conditions as in Figure 3 were applied except that [γ-^33^P]-ATP was used as co-substrate. A stain with coomassie R250 is included as a loding control.

Organisms can inactivate biologically active xenobiotic compounds by a number of reactions including hydrolysis, oxidation or conjugation with metabolites. However, it has also been reported that modification can activate a previously inactive compound [57]. In order to investigated whether the observed modification products of compound 10, namely P1, which according to the previous results represents compound 15, and the two unknown compounds P2 and P3, are potent to inhibit ASKs, we purified the products by solid phase extraction and two fractionations by HPLC. As expected from the previous results (Figure 5 and Additional file 5), the UV spectra of the isolated products P1, P2 and P3 were nearly identical to that of the compounds 10 and 15 (data not shown) indicating that the chromophore, the pyridine ring including the halogen substituent and the amide bond, are still present in the products. This allowed quantification of P1, P2 and P3 by HPLC and UV detection. The potency of the isolated compounds was subsequently investigated by *in vitro* kinase assays (Figure 7). As previously determined, modification product P1 of compound 10 was highly potent to inhibit the kinase activity of BIN2. This was expected since P1 represents compound 15. In contrast, the modification products P2 and P3 had no impact on the activity of BIN2.

A possible modification mechanism that could explain at least one of the additional peaks would be shortening of the side chain by β-oxidation, a pathway which was first recognised by feeding experiments with synthetic ω-phenyl fatty acids [58]. It has also been reported that the plant growth regulator indole-3-butyric acid is activated *in planta* to indole-3-acetic acid (IAA) by a process similar to β-oxidation [59]. To investigate whether P2 or P3 originate from such a process we synthesised compound 17, the expected β-oxidation end product of compound 15 (Additional file 5A). However, the retention time and the UV spectrum of compound 17 were distinct from that of P2 or P3 (Additional file 5B and C), ruling out this possibility.

**Figure 8:**
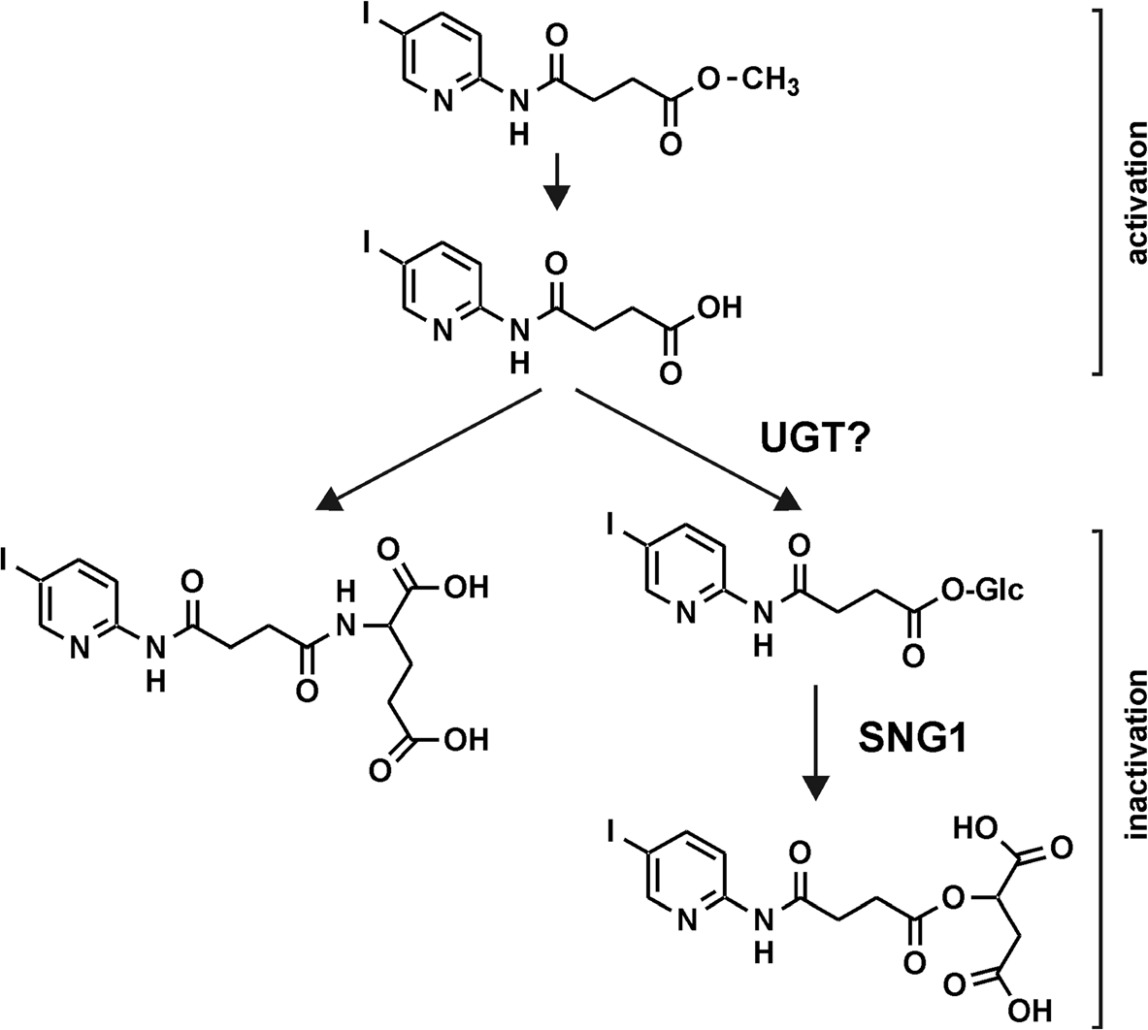
**The catabolic fate of compound 10 in *A. thaliana.*** Compound 10 is rapidly hydrolysed, likely by a lipase, and thereby activated to compound 15. Subsequently, compound 15 is inactivated by conjugation with glutamic acid or with malate. The latter reaction is catalysed by SNG1. Since SNG1 utilises glucosylated substrates compound 15 is presumably glucosylated by a UGT prior to SNG1-mediated conjugation with malate.

To identify the metabolic products of compound 10, we analysed them by high resolution mass-spectrometry. Based on the obtained masses (Table 1) the only possible chemical formula are C_9_H_9_IN_2_O_3_ for P1, C_13_H_13_IN_2_O_7_ for P2 and C_14_H_16_IN_3_O_6_ for P3. This confirms that P1 is nearly identical to compound 15. Since P2 and P3 are formed from P1 (=compound 15; C_9_H_9_IN_2_O_3_) and their UV spectra are identical to that of compound 15, P2 and P3 are presumably the conjugates of compound 15 with glutamic (C_5_H_9_NO_4_) and malic acid (C_4_H_6_O_5_), respectively (Figure 8). The observation of a conjugate with glutamic acid was not surprising since the reaction of amino acids with the carboxyl group of xenobiotics is a widespread reaction in plants [60,61]. To provide additional evidence for the composition of P2 we hydrolysed an aliquot and performed amino acid analysis. In the chromatogram a peak corresponding to glutamic acid was clearly visible (Additional file 6), confirming that P2 is the conjugate of compound 15 with glutamic acid.

**Table 1:** UV and high resolution mass spectrometry

**Compound**	**UV**_**max **_**[nm]**	***m/z*; deviation to the calculated *m/z *** **in ppm; ion type**	**Calculated formula for [M]**
**10**	250/292	334.9892; 1.5; [M + H]^+^	C_10_H_11_IN_2_O_3_
		335.9921; 0.0; [^13^CM + H]^+a^	
		356.9708; 0.3; [M + Na]^+^	
		357.9742; 0.6; [^13^CM + Na]^+^	
		209.0920; 0.5; [M-I + H]^+^	
		210.0954; 0.0; [^13^CM-I + H]^+^	
**15**	250/292	320.9735; 1.2; [M + H]^+^	C_9_H_9_IN_2_O_3_
		321.9764; 0.0; [^13^CM + H]^+^	
		342.9551; 0.3; [M + Na]^+^	
P1	251/292	320.9734; 0.9; [M + H]^+^	C_9_H_9_IN_2_O_3_
		321.9766; 0.6; [^13^CM + H]^+^	
		342.9552; 0.6; [M + Na]^+^	
P2	251/292	450.0163; 1.3; [M + H]^+^	C_14_H_16_IN_3_O_6_
		451.0192; 0.4; [^13^CM + H]^+^	
		471.9978; 0.4; [M + Na]^+^	
		473.0012; 0.4; [^13^CM + Na]^+^	
		324.1192; 0.6; [M-I + H]^+^	
		325.1225; 0.3; [^13^CM-I + H]^+^	
P3	251/292	436.9846; 1.4; [M + H]^+^	C_13_H_13_IN_2_O_7_
		437.9876; 0.5; [^13^CM + H]^+^	
		458.9662; 0.4; [M + Na]^+^	
		459.9695; 0.4; [^13^CM + Na]^+^	
		311.0874; 0.6; [M-I + H]^+^	
		312.0907; 0.6; [^13^CM-I + H]^+^	

^a^[^13^CM] means that the mother molecule contains one ^13^C carbon.

### SNG1 catalyses conjugation of bikinin derivatives with malate

Intriguingly, besides the glutamic acid derivative we could also detect a conjugate of compound 15 with malic acid. While conjugates of xenobiotic compounds with malonic and pyruvic acid are common, conjugates with malic acid are less frequently observed [62] and the pathway for their formation remains elusive.

The structure of the malic acid derivative of compound 15 (Figure 8) resembles that of sinapoylmalate, one of the major soluble phenolic compounds present in *A. thaliana*. Sinapoylmalate is synthesised by glucosylation of sinapic acid by UGT84A2 and its homologues [63] and the subsequent exchange of the glucosyl residue to malate in a reaction catalysed by SNG1 [64]. Because of functional redundancy sinapoylglucose is still formed in *ugt84a2* knock out plants [63]. In contrast, biosynthesis of sinapoylmalate is blocked in *sng1* mutants. To assess whether the malate transferase SNG1 is necessary for generation of P3, we treated *sng1* seedlings with compound 10 and analysed the plant extract by HPLC. While the glutamic acid derivative P2 was still present, the malate derivative P3 was absent in plants deficient in SNG1 function (Figures 5E and F), confirming that SNG1 catalyses formation of the latter. In the chromatogram of the *sng1* plants another peak appeared close to that of P3 while the pronounced peak present at a retention time of 3.5 min in extracts of Col-0 plants (see for instance Figure 5A) was absent. These peaks showed strong fluorescence (Additional file 7) and represent sinapoylglucose (retention time 7.5 min) and sinapoylmalate (retention time 3.5 min). In summary, this result demonstrates that SNG1 catalyses the conjugation of malate with compound 15.

## Discussion

Mutants are widely and successfully used to investigate different biological processes. The application of specific inhibitors is an interesting alternative approach that may offer several advantages compared to mutant analysis. Inhibitors can be used in different genetic backgrounds without the necessity for time consuming crossings. Often they are active in a wide range of organisms allowing investigation of species where no mutants are available. In addition, the application of inhibitors can be limited to a specific developmental stage. This is especially important if a mutant is lethal. For instance, *cyp51* knock out plants die in early seedling stages [65] impeding dissection of the role of CYP51 at later developmental stages. This problem could be circumvented by using the specific CYP51 inhibitor voriconazole, which revealed that CYP51 is highly important for providing precursors for BR biosynthesis [32]. Another remarkable advantage of inhibitors is that they can provide an opportunity to overcome functional redundancy since homologous proteins are often targeted by the same compound. This is especially important for analysis of polyploid organisms but also for gene families. For instance, *A. thaliana* encodes 10 ASKs [17] with several of them involved in BR signalling [16,20]. While dominant gain of function mutants show severe dwarfism [18,19], knock out mutants of single or even several ASKs display no or only very slight phenotypes [21]. In contrast, plants treated with the ASK inhibitor bikinin show the typical signs of constitutive BR response including elongated hypocotyls and light-green leaves [54] indicating that most or all ASKs crucial for BR signalling are inhibited. As a consequence, bikinin has become a widely used and valuable tool for studying BR signalling. Bikinin was originally identified in a chemical genetics screen for compounds mimicking BR responses. *In vitro* kinase assays revealed that it inhibits ASK activity by competing for the kinase binding site with ATP. Docking simulations suggested that the heterocyclic nitrogen may form a hydrogen bond with the backbone amide nitrogen of a conserved valine residue (V118 in BIN2) located in the ATP binding site [54]. This suggests that the heterocyclic nitrogen is crucial for bikinin activity, which is in perfect agreement with our results. Compound 11, which differs from the highly active compound 3 only in the position of the heterocyclic nitrogen, was inactive as revealed by *in vitro* kinase assays. Previous results indicated that the activity of the compounds decreases with the atomic number of the halogen substituent in position 5 of the pyridine ring [54]. However, we found that the fluoro derivative (compound 12) showed little activity to inhibit any ASK while the chloro, bromo and iodo derivatives were highly potent (Figure 3). Interestingly, the inhibitory activity of the compounds on BIN2 increased slightly with the atomic number of the halogen substituent while an opposite although weaker effect was seen for ASKθ (Additional file 2). With respect to that it is important to note that previous docking predictions indicate that the residue interacting with bikinin is a methionine in BIN2 but a leucine in ASKθ, which may explain the different potencies of the chloro, bromo and iodo derivatives for these ASKs.

Analysis of a series of compounds differing only in the length of the aliphatic side chain revealed that the inhibitory effect was highest with a side chain of 4 carbon atoms and a terminal carboxy group (Figure 3). Previous docking simulations indicated that the carboxy group is necessary for interaction with an arginine conserved in the ASKs (arginine R124 in BIN2) [54]. Since the carboxy group of the aliphatic chain of bikinin has a pK_a_ of 5.6 (Additional file 1) it is negatively charged at the intracellular pH. Thus it likely interacts with the positively charged arginine residue by ionic interactions. In agreement with that the methylated derivatives 9 and 10, which are uncharged at a physiological pH, showed little activity *in vitro*. In sharp contrast to *in vitro* assays, the methylated derivatives 9 and 10 were highly active *in vivo* and partially rescued BR mutants. Hypocotyl elongation, a typical response to BRs, was even more strongly induced by compounds 9, 18 and 10 than their free acid counterparts 3, 15 and 14 (Figure 4B and Additional file 3). In addition, dephoshorylation of BZR1, a widely used read out for BR signalling, was induced by the methylated compound 15 to a similar or higher extent than by the free acid compound 10, although only the latter was capable of inhibiting ASKs *in vitro*. The low *in vitro* effectiveness of the methylated inhibitors on the one hand and their high *in vivo* potency on the other hand is likely due to their high cellular permeability and the rapid cleavage of the methyl residue *in planta*, probably by a lipase (peak P1 in Figure 5).

Cellular uptake is a key factor determining inhibitor potency. Tissue permeability assays revealed that uptake of the compounds, especially methylated ones, was rapid. Interestingly, the *in situ* concentrations exceeded that of the surrounding medium several fold. This can be explained by the pK_a_ values of the compounds (Additional file 1). For instance, derivative 15 has a pK_a_ value of 5.8, which means that at pH 5.8, the pH of the medium used, 50% of the compound are dissociated and therefore negatively charged while 50% are undissociated. At the intracellular pH of approximately 7.4 [66] less than 3% of the compound is undissociated. Since only the undissociated, lipophilic form is expected to pass biomembranes efficiently, the compounds are trapped in the cell and accumulate to concentrations exceeding that of the surrounding medium. This pH-dependent uptake resembles that of the plant hormone auxin, where pH-driven diffusion has been suggested to contribute to transport into the cell [67]. The esterified compounds, for instance compound 10, are independent of the pH, highly lipophilic and can pass membranes. In the cell they are rapidly hydrolysed to the corresponding acids, which deprotonate to hydrophilic anions. It is interesting to note that the uptake rate of compound 10 was roughly double that of compound 15, which correlates with the portions capable of diffusion through the membrane: while 100% of compound 10 are lipophilic, only 50% of compound 15 are undissociated and therefore sufficiently lipophilic.

After prolonged incubation the *in situ* levels of compound 15 declined, which was accompanied by the appearance of two peaks designated P2 and P3. The UV spectra of these peaks were nearly identical to that of 15, indicating that they contain the same chromophore, namely the pyridine ring with the amido bond and the halogen substituent. This, together with chemical formulas determined by high resolution mass spectrometry, revealed that P2 and P3 represent the glutamic and malic acid derivatives of compound 15, respectively. While conjugation of xenobiotic compounds with amino acids is a common phenomenon [68], the formation of a conjugate with malic acid was intriguing. The structure of the produced conjugate shows similarity to that of sinapoylmalate and thus we speculated that both compounds might be formed by the same enzyme. Indeed, formation of the malate conjugate of compound 15 was abolished in the *sng1* mutant (Figures 5E and F). SNG1 is a transferase catalysing the formation of sinapolymalate from sinapoylglucose by exchange of the sugar residue for malic acid. This suggests that compound 15 is glucosylated *in planta* prior to conjugation with malic acid (Figure 8) although we could not observe glucosylated compound 15 in the chromatogram. In this respect it is worth noting that sinapoylglucose is hardly detectable in Col-0 plants since, once formed, the glucose residue is immediately exchanged to malate by SNG1, indicating that, in a similar manner to sinapoylglucose, glucosylated compound 15 might be rapidly metabolised.

## Conclusion

The compounds 3, 14 and 15, were highly potent *in vitro* with the iodo derivative (compound 15) showing the highest inhibitory effect on BIN2 and the chloro derivative (compound 3) being most active against ASKθ. *In vivo* the methylated counterparts were more active, since they were significantly better taken up by plants and were immediately hydrolysed *in planta* to the active compounds. Thus, we propose the methylated derivatives of bikinin as inhibitors of choice for *in vivo* studies, especially if a rapid inhibition of ASKs is desired. Bikinin and its derivates are only temporarily active in plants which might be useful to dissect the role of ASK in a defined time window. The inactivation of the inhibitors by conjugation with malate involving SNG1 represents a novel pathway for modification of xenobiotic compounds and opens up new directions for studying the fate of xenobiotics in plants.

## Methods

### Synthesis of the compounds

The compounds were synthesised by reaction of amines with carboxylic acid anhydrides or chlorides using the methods described below. The reaction compounds and yields of the products are listed in Additional file 1.

Method A: A solution of 25 mmol dicarboxylic acid anhydride dissolved in 15 ml tetrahydrofuran (10 ml for phthalic anhydride) was placed in a round bottom flask equipped with a reflux condenser and 20 mmol amine dissolved in 10 ml tetrahydrofuran was added. The mixture was refluxed for 2 hours. The product started to crystallise at the end of the reaction. Crystallisation was completed by cooling to 4°C for several hours. The crude product was filtered with suction and recrystallised from 95% ethanol except for the phthalic acid derivative, which was recrystallised from 80% acetonitrile.

Method B: A solution of 20 mmol 2-amino-5-nitropyridine dissolved in 30 ml tetrahydrofuran was placed in a round bottom flask and 25 mmol solid succinic anhydride was added. A reflux condenser was fitted to the flask and the mixture heated to gentle boiling for 2 hours. Subsequently, the reaction mixture was cooled to −20°C for several days. The crude product was filtered with suction and recrystallised from hot water.

Method C: 20 mmol amine was dissolved in a mixture of 40 ml tetrahydrofuran and 3.5 ml (25 mmol) triethylamine and placed in a triple-necked round bottom flask equipped with a reflux condenser, a dropping funnel and a thermometer. The reaction mixture was agitated by magnetic stirring. A solution of 21 mmol acid chloride dissolved in 10 ml tetrahydrofuran was added slowly through the dropping funnel at such a rate that the temperature did not rise above 40°C. After the chloride had been completely added, the reaction was stirred for further 15 minutes at room temperature. Subsequently, the mixture was poured into 200 ml cold water and the pH set to 6 with diluted hydrochloric acid. The product was extracted three times with 50 ml diethylether and the combined etheral extracts were washed with 50 ml 1% acetic acid. Residual water was removed with anhydrous sodium sulphate prior evaporation of the ether under reduced pressure. The yellowish residue was recrystallised from 95% ethanol (chloro derivatives) or toluene (iodo derivative) to yield an almost white product.

Method D: 21 mmol acid chloride was dissolved in 10 ml tetrahydrofuran and added to a solution of 20 mmol 2-amino-5-chloropyridine in 3.5 ml (25 mmol) triethylamine and 40 ml tetrahydrofuran as described in method C. The mixture was stirred for 15 minutes prior to filtration to remove the triethylamine hydrochloride. The solid was washed with 10 ml tetrahydrofuran and the combined filtrates evaporated under reduced pressure.

In the case of the oxalyl derivate, the residue was dissolved in 90 ml hot 95% ethanol and the solution was filtered while still hot. The mixture was stirred and 40 mmol KOH dissolved in 10 ml water was added at such a rate that the temperature did not rise above 40°C. The reaction was completed by stirring for a further 10 minutes. The product separated as white potassium salt, which was collected by suction. The precipitate was dissolved in 100 ml (iodo derivative: 250 ml) hot water and filtrated. Hydrochloric acid was added to the hot filtrate until reaching pH 2. The product separated as free acid during incubation at 4°C overnight. The product was further purified by recrystallisation from 95% ethanol.

In the case of the malonyl and adipoyl derivatives, the residue was dissolved in 200 ml MeOH and filtrated. The solution was placed in a triple-necked round bottom flask equipped with a reflux condenser, a dropping funnel and a thermometer and heated to 50°C. While stirring the mixture, 40 mmol KOH dissolved in 40 ml water was rapidly added through the dropping funnel while the temperature was maintained at 50°C. The reaction was completed by stirring at the same temperature for an additional 10 minutes. The surplus of KOH was neutralised by the addition of 40 mmol NH_4_Cl dissolved in 10 ml water. Most of the solvent was removed under reduced pressure and the residue was dissolved in water (about 200 ml) and filtered. Formic acid was added to the clear filtrate until reaching pH 3. The product separated as white crystals during incubation at 4°C overnight. The malonyl and adipoyl derivatives were purified by recrystallisation from 95% or 50% ethanol, respectively.

### Determination of pK_a_ values

Fifty to 100 mg of each compound were weighed and dissolved in 50 ml 50% (v/v) methanol. A titration curve with a 50 mM NaOH standard solution was recorded with a Greisinger Electonics GPHR 1400A pH meter. The equivalence point was determined by the difference quotient method (ΔpH/ΔV_NaOH_) and the pK_a_ read from the titration curve at 50% neutralisation.

### *In vitro* kinase assays

ASKs were expressed as GST-fusion proteins in *Escherichia coli* BL21 as described previously [55]. *In vitro* kinase assays were performed by incubating 50 ng GST-fusion protein, 10 μg myelin basic protein (MBP; Sigma, St Louis, MO) as a substrate and 0.15 MBq γ-[^32^P]-ATP as co-substrate at 25°C for 30 min. The reaction buffer consisted of 20 mM HEPES/KOH pH 7.4, 15 mM MgCl_2_, 5 mM EGTA, 1 mM dithiothreitol and 1 μM cold ATP. The reaction products were separated by SDS-PAGE and the amount of radioactivity incorporated into MBP quantified using an Amersham storage phosphor imager screen and a Biorad Molecular Imager FX.

### Physiological tests

*Arabidopsis thaliana* Col-0 or *bri1-1* seedlings were grown aseptically on ½ Murashige and Skoog (MS) plates containing 0.8% agar and 1% sucrose in a growth camber under long day conditions (16 h cool white fluorescent light at a photon flux of 80 μmol · m^−2^ · s^−1^, 8 h dark) at 21°C for 7 days. Subsequently, they were transferred to plates supplemented with inhibitors in different concentrations and effects on the phenotype were observed 7 days later. For hypocotyl measurements Col-0 seeds were plated on ½ MS plates containing the indicated compounds, incubated at 4°C in the dark for 2 day for stratification and subsequently transferred to long day conditions. After 6 day hypocotyl length was measured using an Olympus SZX10 microscope at 10-fold magnification.

### Generation of lines transgenic for CFP tagged versions of BZR1 or BES1

*A. thaliana* plants were transformed with the constructs pGWR8-BZR1-CFP or pGWR8-BES1-CFP [20] by the floral dip method [56]. For each transgene one line with a representative subcellular BZR1 or BES1 expression pattern was selected from at least 20 independent lines.

### Western blot analysis

Plants transgenic for BZR1-CFP or BES1-CFP grown for 10 day on ½ MS medium containing 1% sucrose were transferred to liquid medium and treated with the indicated compounds for 2 h. Harvested samples (50 mg) were shock-frozen in liquid nitrogen, homogenized with a Retsch mill and after addition of 200 μl extraction buffer (62.5 mM TRIS pH 6.8, 125 mM DTT, 2.5% SDS, 12.5% glycerol, 0.01% bromophenol blue) immediately incubated at 95°C for 2 min. The samples were centrifuged at 15000 g for 5 min and 10 μl of the supernatants separated by SDS-PAGE (10% gel) and blotted onto a polyvinylidene difluoride membrane (Millipore, Billerica, MA). The membrane was blocked with blocking buffer (5% skim milk powder dissolved in 0.05% Tween 20, 150 mM NaCl and 10 mM TRIS/HCl pH 8.0) and subsequently probed with anti-GFP antibody (Roche, Basel, Switzerland) diluted 1:5000 in blocking buffer. Alkaline phosphatase-conjugated goat anti-mouse Fab specific fragment (Sigma-Aldrich, St. Louis, MO, USA) diluted 1:5000 with blocking buffer was employed as secondary antibody. For detection the CDP-Star™ detection reagent (GE Healthcare, Buckinghamshire, UK) was used.

Protoplasts were transformed as described previously [57] and treated with the indicated compounds for 1 h. After centrifugation, the protoplasts were lysed by adding 10 μl 4× SDS loading buffer to 30 μl protoplast suspension and immediately heated to 95°C for 2 min. Western blot analysis was performed as described above except that 20 μl protein extract were used for SDS-PAGE.

### Analysis of plant extracts by HPLC

Two-week-old *A. thaliana* Col-0 seedlings were vacuum infiltrated with ½ MS or ½ MS containing 50 μM compound 10 as described previously [58]. Samples were taken at the indicated time points, rinsed with water and ground in liquid nitrogen to a fine powder. 100 mg powder was weighed into a reaction tube and 1 ml extraction buffer (20% acetonitrile (ACN) containing 20 mM TRIS/HCl pH 6.8) was added. After incubation for 30 min in a shaker set to 800 rpm, the mixture was centrifuged and the supernatant passed through a 0.2 μm filter and analysed by HPLC. The HPLC system comprised a Dionex P680 pump, an ASI-100 autosampler and a PDA-100 photodiode array detector. The system was equipped with a 250 × 4 mm Nucleosil 100–5 C_18_ 250 × 4 mm column (Macherey-Nagel, Düren, Germany) preceded by a Valco 2 μm inline-filter. A constant flow rate of 1 ml/min was maintained with a gradient of solvent A (20 mM acetic acid set to pH 4.8 with NaOH in 15% ACN) and solvent B (20 mM acetic acid set to pH 4.8 with NaOH in 60% ACN). Elution began with an isocratic flow of solvent A for 1 min. The concentration of solvent B was then raised linearly to 100% within 19 min and kept isocratic for another 2 min prior to reducing it to 0% within 1 min. The column was equilibrated for 5 min with solvent A before injection of the next sample. The UV spectra were recorded from 220 to 400 nm with 1 nm intervals. For quantification the absorbance at 250 nm with a bandwidth of 10 nm was recorded.

### Cell-permeability assay

Two-week-old *A. thaliana* Col-0 seedlings were transferred to liquid ½ MS medium containing 50 μM inhibitor. Samples were removed after the indicated time points, rinsed twice with water, dried with filter paper and frozen in liquid nitrogen. For analysis the plant material was ground to a fine powder in a mortar pre-cooled with liquid nitrogen. Approximately 100 mg powder was weighed into 1.5 ml reaction tubes and 1 ml 20 mM TRIS/HCl pH 8.0 added. 50 μl of a 200 μM stock of compound 4 was added as internal standard. Extraction was performed at 80°C for 30 min in an Eppendorf thermo mixer set to 800 rpm. The extract was centrifuged for 5 min at 15000 g and the clear supernatant was collected. The clear solution was acidified by addition of 25 μl 4 M phosphoric acid and centrifuged for 2 min at 15000 g. The supernatant was loaded immediately onto a PH 100 mg solid-phase-extraction cartridge (Varian, Lake Forest, CA) conditioned with 1 ml ACN and two times 1 ml 100 mM phosphoric acid. Columns were washed with 1 ml 100 mM phosphoric acid and dried by applying vacuum for 1 min. Subsequently, elution was performed with 1 ml 100 mM TRIS/HCl pH 9.0 containing 5% ACN. The eluate was acidified by addition of 15 μl 4 M phosphoric acid and used for HPLC as described above.

### Isolation of modified compounds

Two-week-old *A. thaliana* Col-0 seedlings (600 mg) were transferred to liquid ½ MS medium and compound 15 was added to a final concentration of 50 μM. After 48 h incubation the plant material was collected, ground in liquid nitrogen to a fine powder and extracted as described above. After centrifugation the clear supernatant was acidified with 37.5 μl concentrated formic acid and loaded on a 500 mg LiCrolute RP-18 conditioned with 3 ml ACN and 3 ml water. The column was washed with 3 ml water and eluted with 3 ml 50 mM TRIC/HCl pH 8.0 in 20% ACN. The eluate was evaporated in the vacuum to dryness, dissolved in 200 μl water and injected into a HPLC system equipped with a Nucleosil 100–5 C18 200 × 4.6 mm column (Macherey-Nagel, Düren, Germany) preceded by a C18 4 × 3 mm Security Guard Cartridge (Phenomex, Aschaffenburg, Germany). Elution was started with an isocratic flow of 1 ml/min of solvent A (20 mM acetic acid set to pH 4.8 with NaOH in 15% ACN) for 1 min before the concentration of solvent B (20 mM acetic acid set to pH 4.8 with NaOH in 60% ACN) was raised linearly to 60% over 19 min. Fractions were collected every 0.5 min and analysed for the presence of modified compounds by UV spectroscopy. Fractions containing the desired compound were pooled, evaporated to dryness and the residue was dissolved in 200 μl water and injected into the same HPLC system described above except that elution was performed with a linear gradient of 90% solvent A (0.1% acetic acid in water) and 10% solvent B (80% ACN in water) to 60% A and 40% B within 20 min at a constant flow rate of 1 ml/min. Fractions were collected and processed in the same way as described above. After evaporation the compounds were dissolved in 20 mM TRIS/HCl pH 8.0. The concentrations were quantified by HPLC as described above using compound 15 as a standard and compound 14 as internal standard.

### Liquid chromatography-high resolution mass spectrometry

Collected eluates were separated by HPLC using a Hypersil Gold C18 3 μm 150 × 2.1 mm column (Thermo Fischer Scientific, Vienna, Austria) preceded by a C18 4 × 2 mm guard column (Phenomex, Aschaffenburg, Germany). A constant flow rate of 1 ml/min was maintained with a gradient with eluent A (0.1% formic acid in water) and eluent B (0.1% formic acid in ACN). Elution started isocratic with 90% A and 10% B for one minute. Subsequently, B was linearly raised to 100% within 19 min and then kept at 100% for further 2 minutes prior reducing it to the starting conditions within 0.1 min. The column was finally equilibrated for 7.9 min with 90% A and 10% B before the next sample was injected. For detection a LTQ Orbitrap XL high-resolution mass spectrometer (Thermo Fisher Scientific) was used. n-BBS (n-butylbenzenesulfonamide) was constitutively present in our system and was used as lock-mass ([n-BBS + H]^+^; *m/z* of 214.08963). Chemical formulas were calculated with Xcalibur 2.1.1 with the following numbers of atoms: C: 0–30; H: 0–60; O: 1–10; N: 1–10; I: 0–2.

### Amino acid analysis

Compound P2 was hydrolysed with 6 M hydrochloric acid at 110°C for 4 h. The amino acid composition in the hydrolysate was analysed as described previously [59].

## Competing interests

Claudia Jonak and Wilfried Rozhon are listed as inventors on a patent related to the work described in this study, which is held by the GMI-Gregor Mendel Institute of Molecular Plant Biology.

## Authors’ contribution

WR conceived the study and was involved in all stages of experimental work and data analysis and drafted the manuscript. WW analysed the impact of the compounds on the hypocotyl growth and performed the western blot analysis of stably transformed plants and the kinase assay with the isolated conjugates. FB performed the liquid chromatography-high resolution mass spectrometry analysis. JM and EP participated in construct cloning, plant transformation and selection of transgenic lines. TC measured the IC_50_ values and performed the amino acid analysis. TS and BP participated in data analysis. CJ participated in data analysis and drafted the manuscript. All authors read and approved the final manuscript.

## Supplementary Material

Additional file 1 Overview about the methods used for synthesis of the compounds. Click here for file

Additional file 2 Impact of the halogen substituent on the potency of the compounds. Click here for file

Additional file 3 Hypocotyl elongation assays with compounds 3, 9, 14 and 18. Click here for file

Additional file 4 Inhibitory effect of compounds 10 and 15 in the protoplast system. Click here for file

Additional file 5 Comparison of retention times and UV spectra of the conjugates P1, P2 and P3 with standards. Click here for file

Additional file 6 Amino acid analysis of conjugate P2. Click here for file

Additional file 7 **Identification of plant metabolites appearing in the chromatograms of Figure **5**.** Click here for file
