# Supplementary material for: Bikinin-like inhibitors targeting GSK3/Shaggy-like kinases: characterisation of novel compounds and elucidation of their catabolism in planta
Source: BMC Plant Biol. 2014 Jun 19;14:172. doi: 10.1186/1471-2229-14-172 (PMC4078015; doi:10.1186/1471-2229-14-172)
Supplement: Additional file 1 — Overview about the methods used for synthesis of the compounds. [file 1471-2229-14-172-S1.pdf]

Additional file 1: Compounds synthesised

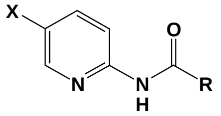

Compounds 1-10, 12-18

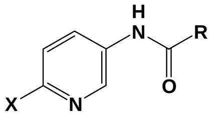

Compound 11

No.	Reaction compounds	Method	X	R	Yield	λ_1; λ_2[a]	pK_a[b]
**1**	2-Amino-5-chloropyridine, Methyl oxalyl chloride	D	Cl	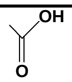	39%	254; 291	
**2**	2-Amino-5-chloropyridine, Methyl malonyl chloride	D	Cl	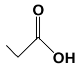	30%	245; 287	
**3**	2-Amino-5-chloropyridine, Succinic anhydride	A	Cl	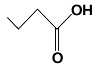	54%	245; 287	5.4
**4**	2-Amino-5-chloropyridine, Glutaric anhydride	A	Cl	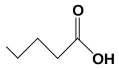	71%	245; 288	
**5**	2-Amino-5-chloropyridine, Methyl adipoyl chloride	D	Cl	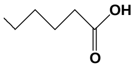	20%	245; 288	
**6**	2-Amino-5-chloropyridine, Maleic anhydride	A	Cl	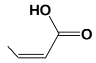	22%	222; 298	
**7**	2-Amino-5-chloropyridine, Phthalic anhydride	A	Cl	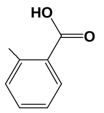	63%	249; 289	
**8**	2-Amino-5-chloropyridine, Methyl malonyl chloride	C	Cl	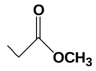	41%	245; 287	
**9**	2-Amino-5-chloropyridine, Methyl succinyl chloride	C	Cl	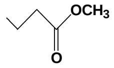	33%	245; 288	
**10**	2-Amino-5-iodopyridine, Methyl succinyl chloride	C	I	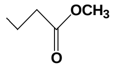	30%	252; 293	
**11**	5-Amino-2-chloropyridine, Succinic anhydride	A	Cl	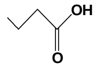	69%	248; 284	
**12**	2-Aminopyridine, Succinic anhydride	A	H	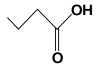	74%	235; 276	4.9
**13**	2-Amino-5-fluoropyridine, Succinic anhydride	A	F	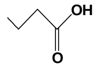	68%	235; 283	
**14**[c]	2-Amino-5-bromopyridine, Succinic anhydride	A	Br	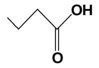	65%	247; 289	5.6
**15**	2-Amino-5-iodopyridine, Succinic anhydride	A	I	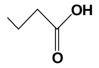	35%	252; 292	5.8
**16**	2-Amino-5-nitropyridine, Succinic anhydride	B	NO_2	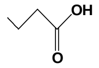	29%	221; 350	
**17**	2-Amino-5-iodopyridine, Methyl oxalyl chloride	D	I	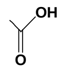	28%	253; 293	
**18**	2-Amino-5-bromopyridine, Methyl succinyl chloride	C	Br	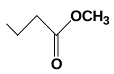	39%	247; 289	

[a] Absorption maxima in nm (measured in 15% ACN containing 20 mM sodium acetate buffer pH 4.8).
[b] The pK_a (negative decadic logarithm of the dissociation constant) of the carboxylic acid group was determined in 50% (v/v) methanol due to limited solubility of the compounds in water.
[c] Compound **14** is bikinin.
