# Supplementary material for: Bikinin-like inhibitors targeting GSK3/Shaggy-like kinases: characterisation of novel compounds and elucidation of their catabolism in planta
Source: BMC Plant Biol. 2014 Jun 19;14:172. doi: 10.1186/1471-2229-14-172 (PMC4078015; doi:10.1186/1471-2229-14-172)
Supplement: Additional file 2 — Impact of the halogen substituent on the potency of the compounds. [file 1471-2229-14-172-S2.pdf]

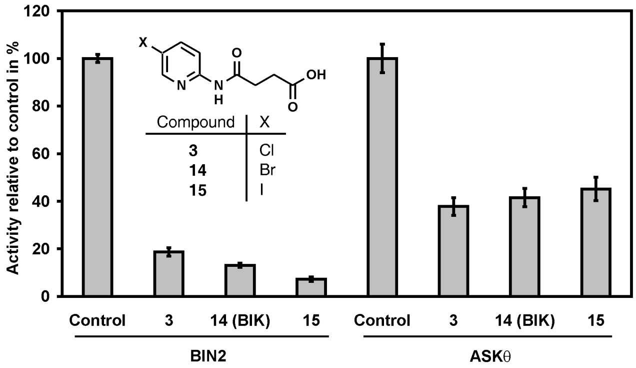


**Additional file 2**: Impact of the halogen substituent on the potency of the compounds. GST-BIN2 or GST-ASKθ was incubated with MBP as a substrate and [γ-$^{32}$P]-ATP as a co-substrate in absence (control) or presence of compounds **3**, **14** (BIK, bikinin) and **15** at a concentration of 10 µM. The proteins were separated by SDS-PAGE and phosphorylation of MBP was quantified using a phosphor imager screen. The residual activity is expressed in % of the corresponding control. The means and standard deviations were calculated from 4 independent assays. The insert shows the structures of the tested compounds.
