# Supplementary material for: Bikinin-like inhibitors targeting GSK3/Shaggy-like kinases: characterisation of novel compounds and elucidation of their catabolism in planta
Source: BMC Plant Biol. 2014 Jun 19;14:172. doi: 10.1186/1471-2229-14-172 (PMC4078015; doi:10.1186/1471-2229-14-172)
Supplement: Additional file 3 — Hypocotyl elongation assays with compounds 3, 9, 14 and 18. [file 1471-2229-14-172-S3.pdf]

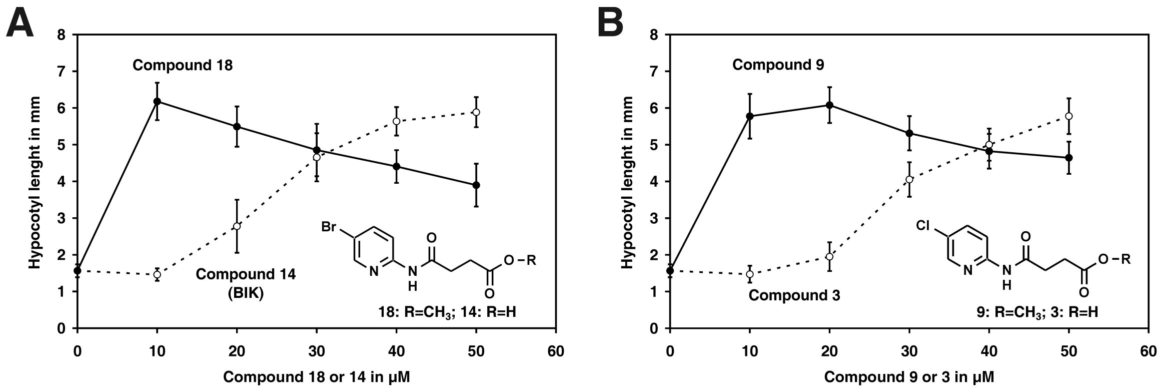

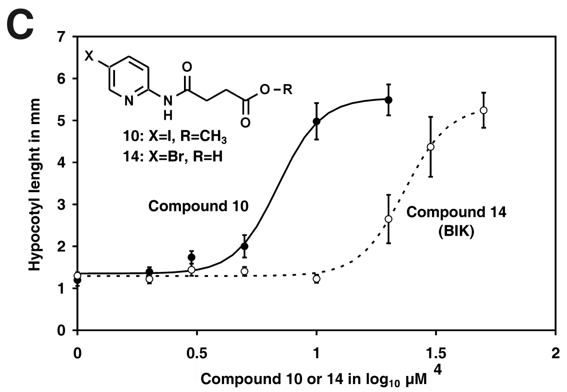

4

Logistic function:

$$y = min + \frac{max - min}{1 + 10^{b * (\log_{10} IC_{50} - x)}}$$

Regression parameters:

	Compound 10	Compound 14
min	1.352 mm	1.292 mm
max	5.536 mm	5.339 mm
b	4.885 $\log_{10}$ mm/$\log_{10}$ µM	4.710 $\log_{10}$ mm/$\log_{10}$ µM
IC_{50}	6.911 µM	23.288 µM

Additional file 3: Effect of methylated and unmethylated derivatives on hypocotyl length. (A) The hypocotyl length of 7-day-old *A. thaliana* Col-0 seedlings grown on ½ MS medium containing 1% sucrose and supplemented with compound **18** or **14** (BIK, bikinin) at the indicated concentrations was measured. (B) The same experimental setup as in (A) was used to test compounds **9** and **3**. The means and standard deviations were calculated from at least 22 seedlings. (C) Comparison of compound **10** and **14** (BIK, bikinin). The hypocotyl length of plants grown on different concentrations of both concentrations was measured as described above. The data were fitted using a logistic function (solid and dotted line) and the IC_{50} values were calculated.
