# Supplementary material for: Bikinin-like inhibitors targeting GSK3/Shaggy-like kinases: characterisation of novel compounds and elucidation of their catabolism in planta
Source: BMC Plant Biol. 2014 Jun 19;14:172. doi: 10.1186/1471-2229-14-172 (PMC4078015; doi:10.1186/1471-2229-14-172)
Supplement: Additional file 4 — Inhibitory effect of compounds 10 and 15 in the protoplast system. [file 1471-2229-14-172-S4.pdf]

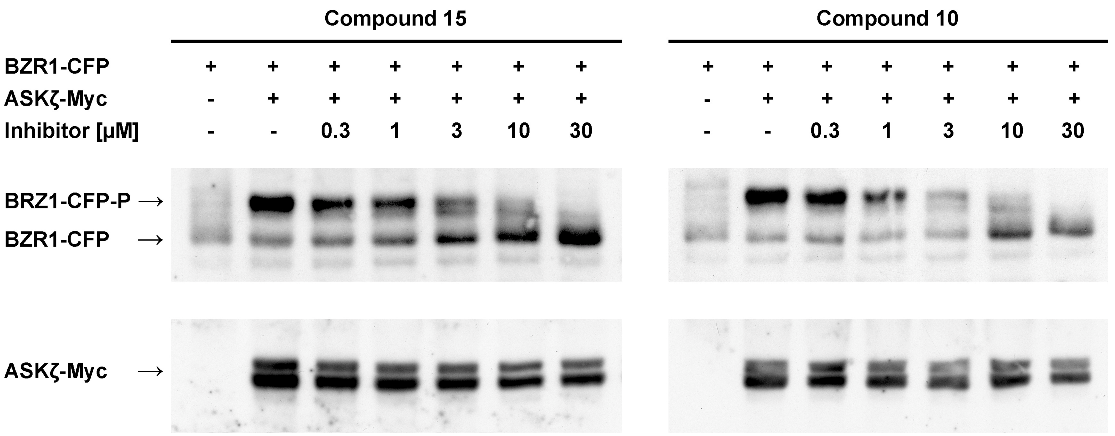

Additional file 4: Compounds **10** and **15** are potent inhibitors *in vivo*. *A. thaliana* protoplasts were co-transformed with expression constructs for BZR1-CFP and Myc-tagged ASKζ and treated with compounds **10** and **15** at the indicated concentrations. BZR1-CFP and ASKζ-Myc were detected by western blot analysis using monoclonal anti-GFP and anti-Myc antibodies, respectively. Depending on the ASKζ kinase activity BZR1-CFP occurs in a phosphorylated or unphosphorylated form (indicated by arrows). ASKζ-Myc appeared as two bands indicating posttranslational modification of this protein. ASKζ was selected for this experiment since BIN2 is not expressed in the protoplast system.
