# Supplementary material for: Bikinin-like inhibitors targeting GSK3/Shaggy-like kinases: characterisation of novel compounds and elucidation of their catabolism in planta
Source: BMC Plant Biol. 2014 Jun 19;14:172. doi: 10.1186/1471-2229-14-172 (PMC4078015; doi:10.1186/1471-2229-14-172)
Supplement: Additional file 5 — Comparison of retention times and UV spectra of the conjugates P1, P2 and P3 with standards. [file 1471-2229-14-172-S5.pdf]

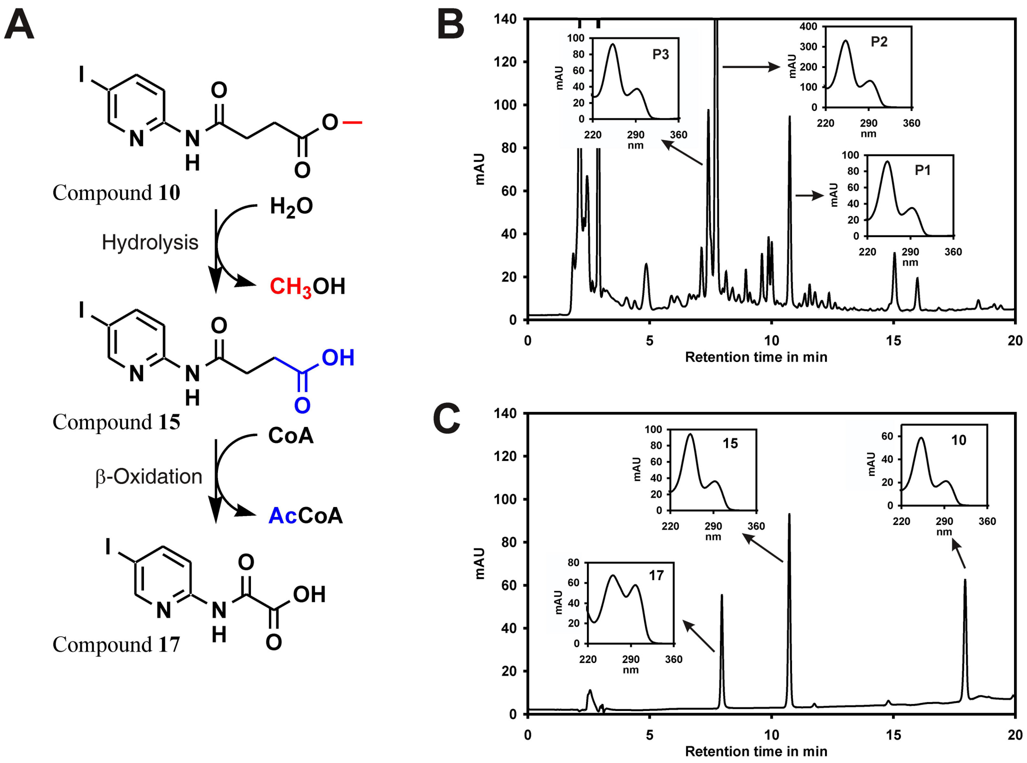

Additional file 5: Products P2 or P3 are not derived by β-oxidation. (A) Proposed pathway for degradation of compound **10** by β-oxidation. Compound **10** is first hydrolysed to compound **15**, which undergoes shortening of the aliphatic side chain by β-oxidation to finally yield compound **17**. However, this pathway is not supported by experimental data: (B) Chromatogram of a cell extract derived from Col-0 seedlings treated with 50 µM compound **15**. The UV spectra of the obtained products are shown in the inserts. (C) Chromatogram of a mixture of synthesised compounds **10**, **15** and **17**, the latter representing the putative β-oxidation end product. As indicated by the retention times and the UV spectra P1 is identical to compound **15** while P2 and P3 are distinct from compound **17**.
