# Supplementary material for: Bikinin-like inhibitors targeting GSK3/Shaggy-like kinases: characterisation of novel compounds and elucidation of their catabolism in planta
Source: BMC Plant Biol. 2014 Jun 19;14:172. doi: 10.1186/1471-2229-14-172 (PMC4078015; doi:10.1186/1471-2229-14-172)
Supplement: Additional file 6 — Amino acid analysis of conjugate P2. [file 1471-2229-14-172-S6.pdf]

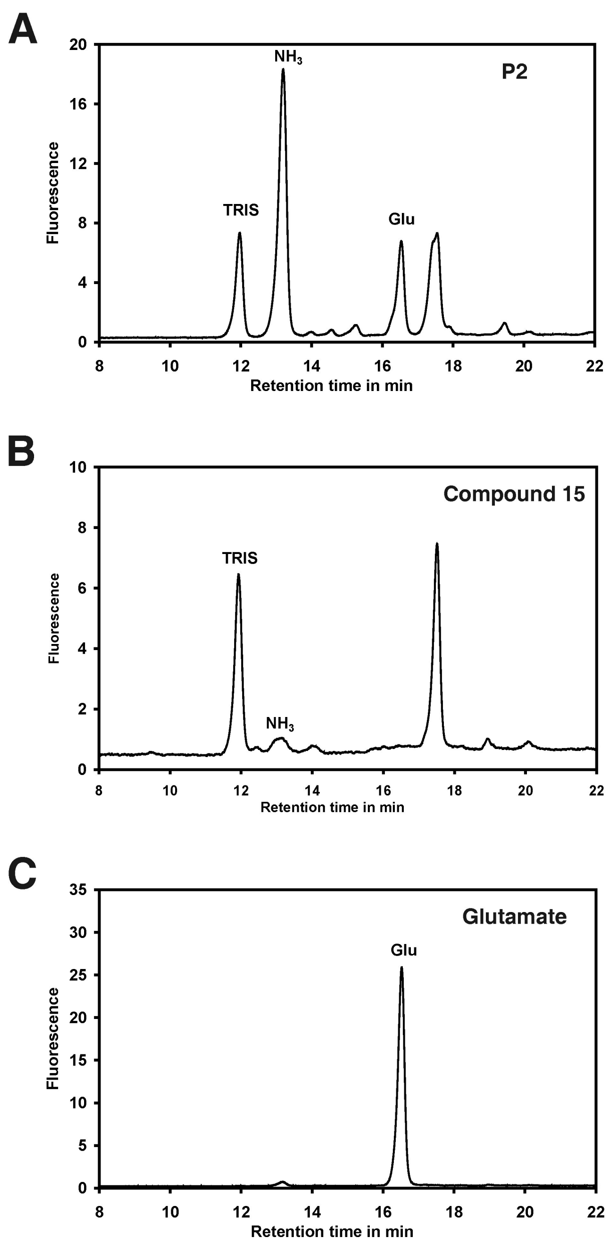

Additional file 6: Amino acid analysis of P2. (A) An aliquot of the purified product was hydrolysed with hydrochloric acid, the hydrolysis products derivatised with dansyl chloride and analysed by HPLC. (B) The same analysis was performed with compound **15**, the precursor of P2. (C) Glutamic acid derivatised with dansyl chloride. The peak for TRIS originates from the buffer used to dissolve P2 and compound **15**. Glutamate was dissolved in pure water and thus this peak is absent in (C). During acidic hydrolysis ammonia is formed, causing the peak at a retention time of 15 min. The peak at 16.5 min represents glutamic acid and is only visible in P2 (A) and the glutamic acid control (C) but absent in the chromatogram of compound **15** (B). The peak with a retention time of 17.6 min represents a cleavage product of compound **15**, likely 2-aminopyridine. The expected product, 2-amino-5-iodipyridine, is not visible, suggesting that it is dehalogenated under the harsh conditions required for hydrolysis. The fluorescence was recorded at an excitation wavelength of 355 nm and an emission wavelength of 515 nm.
