# Supplementary material for: Bikinin-like inhibitors targeting GSK3/Shaggy-like kinases: characterisation of novel compounds and elucidation of their catabolism in planta
Source: BMC Plant Biol. 2014 Jun 19;14:172. doi: 10.1186/1471-2229-14-172 (PMC4078015; doi:10.1186/1471-2229-14-172)
Supplement: Additional file 7 — Identification of plant metabolites appearing in the chromatograms of Figure 5. [file 1471-2229-14-172-S7.pdf]

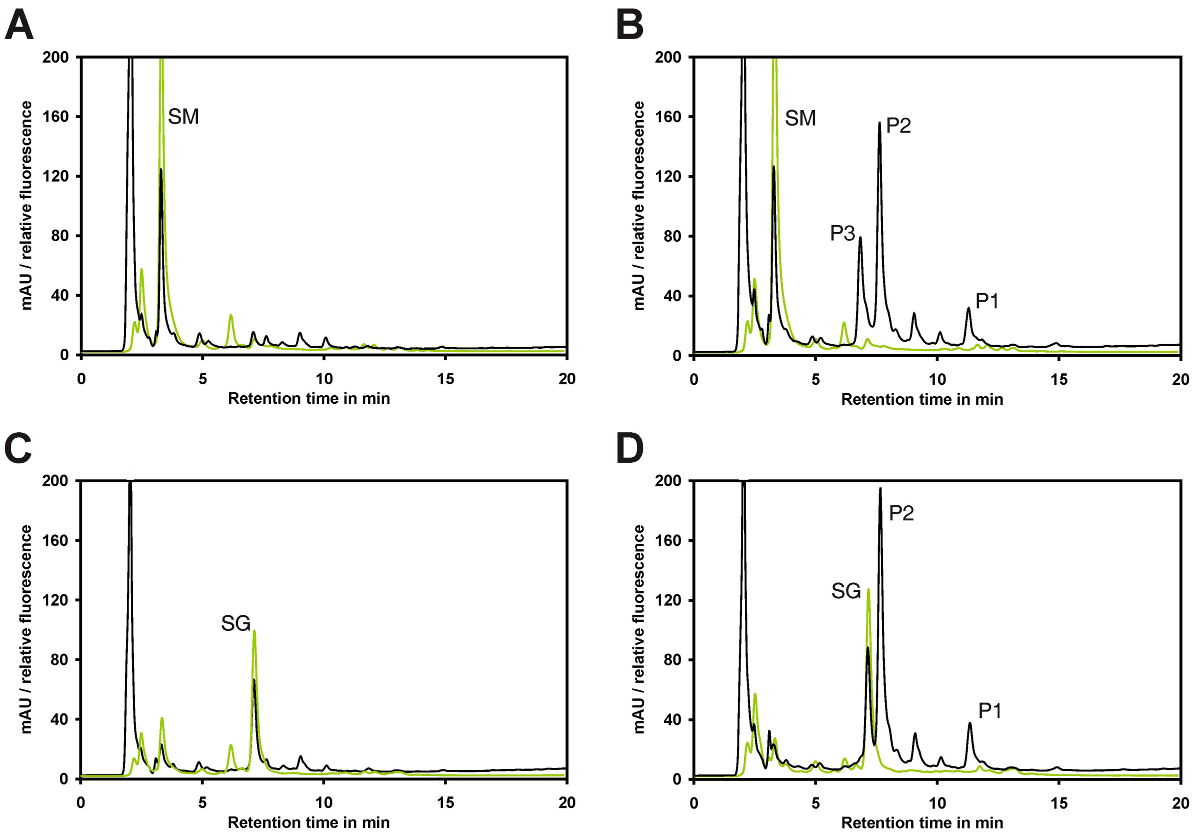

Additional file 7: Detection of sinapoylmalate (SM) and sinapoylglucose (SG) by HPLC. Chromatograms of extracts derived from (A) untreated Col-0, (B) Col-0 treated with 50 µM compound **10**, (C) untreated *sng1* and (D) *sng1* treated with 50 µM compound **10**. For detection the absorbance at 250 nm (black line) and the fluorescence (λ_{Ex}: 335 nm/λ_{Em}: 450 nm; green line) was recorded.
